# Surgical vs. trans-catheter aortic valve replacement in patients With bicuspid aortic valve stenosis

**DOI:** 10.3389/fcvm.2026.1871325

**Published:** 2026-06-25

**Authors:** Kobi Faierstein, Tal Caller, Dassi Moshkovitz, Efrat Sharon, Roy Raphael, Ido Cohen, Noam Makmal, Efrat Mazoe Dray, Afek Ash, Elad Maor, Ehud Regev, Amit Segev, Israel M. Barbash, Paul Fefer

**Affiliations:** 1 Leviev Cardiovascular Center, Sheba Medical Center, Ramat Gan, Israel; 2 Gray Faculty of Medical and Health Sciences, Tel-Aviv University, Tel-Aviv, Israel; 3 Neufeld and Tamman Cardiovascular Research Institutes, School of Medicine, Tel Aviv University, Ramat-Gan, Israel

**Keywords:** bicuspid aortc valve, heart failure hospitalization, mortality, pacemaker (PM), paravalular leak (PVL), SAVR, stroke, TAVI

## Abstract

**Background:**

Aortic valve stenosis can be treated surgically or with transcatheter aortic valve implantation (TAVI). However, bicuspid aortic valve (BAV) anatomy presents unique challenges that may affect procedural outcomes. We aimed to compare outcomes between the two approaches.

**Methods:**

a retrospective cohort of 480 patients with BAV stenosis: 75 (16%) underwent TAVI and 405 (84%) underwent SAVR. Median age was 76 (Q1–Q3: 72–82) in the TAVI group and 61 (Q1–Q3: 52–70) in the SAVR group. Patients with isolated aortic insufficiency were excluded. Primary analyses were adjusted for age, sex, and comorbidities, followed by a sensitivity analysis using 1:1 age matching. Outcomes included overall survival, stroke/TIA, pacemaker implantation, paravalvular leak, and heart failure hospitalizations.

**Results:**

During a mean follow-up of 9 ± 5 years, 72 patients died (28 TAVI, 44 SAVR). TAVI was associated with higher mortality (adjusted HR = 5.9, 95% CI 2.6–12.9, *p* < 0.001), higher rates of pacemaker implantation (19% vs. 4%; HR = 2.6, 95% CI 1.1–6.7, *p* < 0.001), paravalvular leak (adjusted OR = 5.8, 95% CI 2.6–12.7, *p* < 0.001) and heart failure hospitalizations (IRR=7.7, 95% CI 2.7–22.2, *p* < 0.001). Stroke rates were similar. Age-matched analyses confirmed higher mortality (adjusted HR = 3.6, 95% CI 1.5–8.6, *p* = 0.004), increased pacemaker implantation (adjusted HR = 3.9, 95% CI 1.04–14.5, *p* = 0.002), and heart failure hospitalizations (IRR=7.7, 95% CI 3.9–15.1, *p* < 0.001).

**Conclusions:**

In BAV stenosis, SAVR was associated with better survival and fewer complications than TAVI, while stroke/TIA rates were similar. Randomized trials are needed to determine the optimal treatment approach.

## Graphical Abstract

**Figure:**
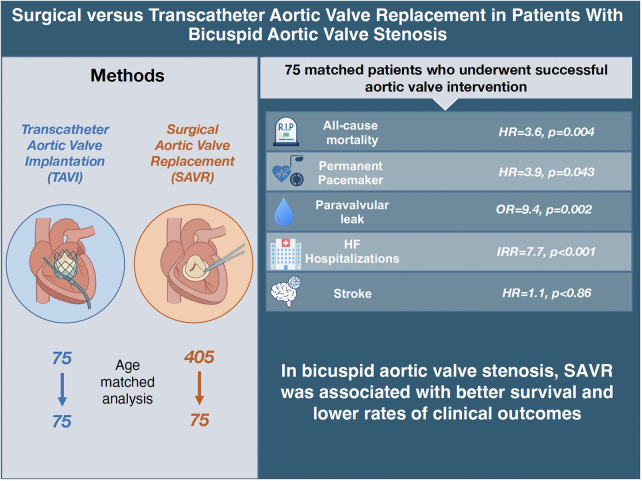

## Introduction

Bicuspid aortic valve (BAV) is the most common congenital cardiac anomaly, affecting approximately 1%–2% of the population, and is a frequent cause of premature aortic stenosis. Historically, surgical aortic valve replacement (SAVR) has been the standard of care in this population. With the expanding use of transcatheter aortic valve implantation (TAVI) in lower-risk and younger patients, there is growing interest in evaluating its safety and efficacy in BAV anatomies. Randomized controlled trials such as PARTNER 3 and Evolut Low Risk have established TAVI as a safe and effective therapy for tricuspid aortic valve stenosis in low and intermediate risk patients (1–3). However, BAV patients were largely excluded from these pivotal studies. More recent observational analyses have reported acceptable short and mid-term outcomes of TAVI in selected BAV anatomies (3, 4). However, due to the anatomical complexity and calcification patterns associated with BAV, concerns persist regarding procedural outcomes such as paravalvular leak and conduction disturbances, while long-term durability remains uncertain. Direct comparisons between TAVI and SAVR in BAV patients remain limited. The aim of this study was to compare clinical outcomes, including all-cause mortality, heart failure hospitalizations, paravalvular leaks, permanent pacemaker implantations and stroke, of patients with BAV stenosis who underwent TAVI vs. SAVR at a large tertiary medical center.

## Methods

### Study population, data sources, and inclusion/exclusion criteria

**Figure 1:**
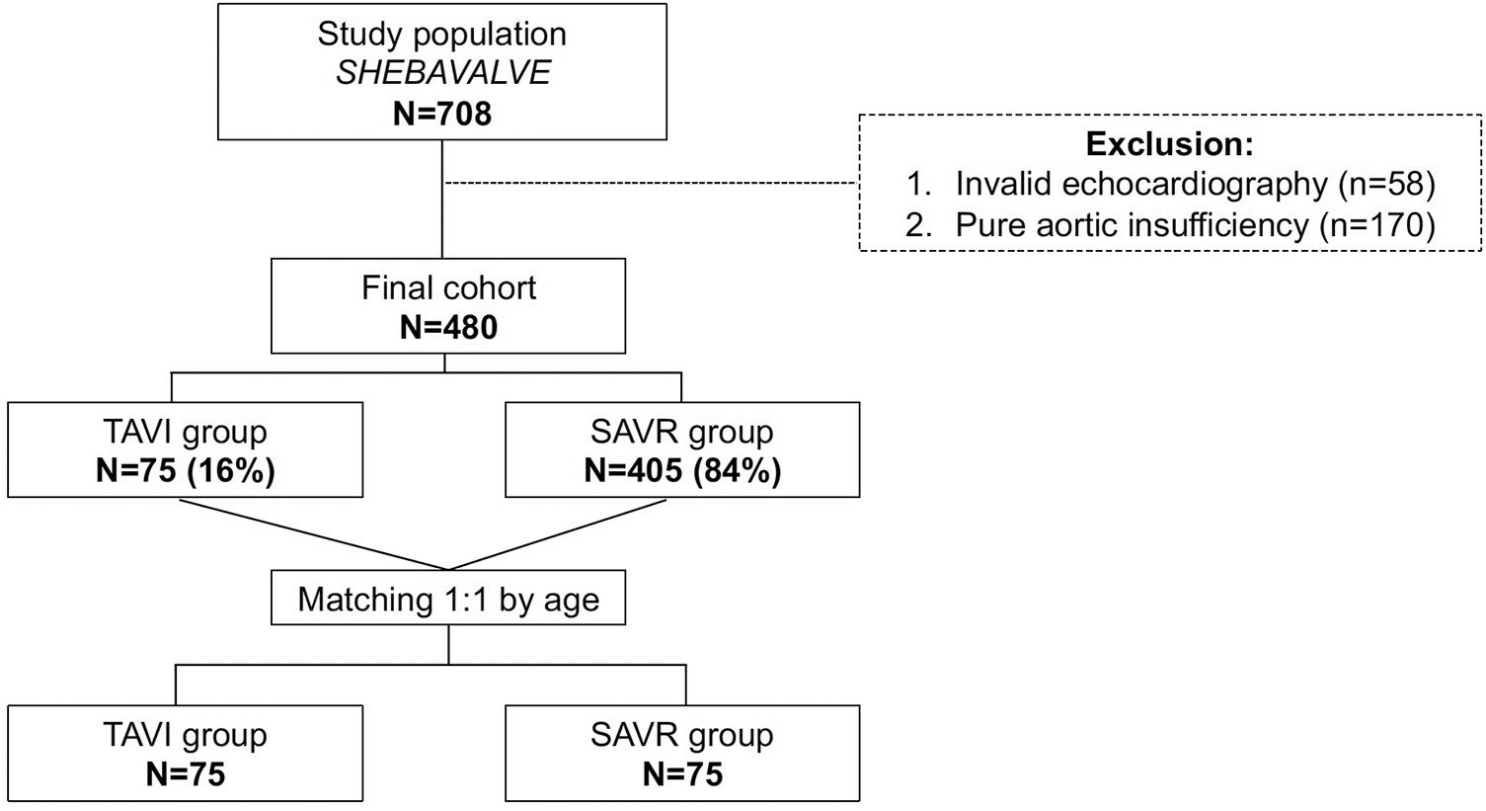
Study Flowchart.

This retrospective study included patients with bicuspid aortic valve (BAV) stenosis who underwent transcatheter aortic valve implantation (TAVI) or surgical aortic valve replacement (SAVR) at Sheba Medical Center, the largest tertiary medical center in Israel. All patients with aortic stenosis at our institution are evaluated in a dedicated valvular clinic and discussed in a multidisciplinary Heart Team meeting, where the decision regarding TAVI or SAVR is made. The initial dataset included all patients aged >18 years with BAV stenosis who underwent TAVI or SAVR between 2008 and 2024. Patients with isolated aortic regurgitation or missing echocardiographic data were excluded. The final cohort consisted of two groups: patients with BAV stenosis treated with TAVI and those treated with SAVR. Overall, 480 adult patients were included (Figure 1).

### Study endpoints and definitions

The primary endpoint was all-cause mortality during follow-up. Secondary endpoints included: (1) permanent pacemaker implantation within 3 months after the procedure, (2) stroke or transient ischemic attack (TIA) during follow-up, (3) heart failure (HF) hospitalizations after the procedure, calculated as yearly incidence and expressed as an incidence rate ratio (IRR) between groups, and (4) paravalvular leak (PVL), defined as moderate or greater regurgitation on follow-up echocardiography within 6 months post-procedure. Survival data were obtained from the Israeli Population Registry. The Institutional Review Board of Sheba Medical Center approved the study with strict maintenance of participant anonymity during database analyses; therefore, individual consent was waived.

### Statistical analysis

Continuous variables are presented as mean ± standard deviation (SD), and categorical variables as frequencies and percentages. Follow-up was calculated from the procedure date until death or end of follow-up. Kaplan–Meier curves were used to compare survival between the TAVI and SAVR groups. Univariable and multivariable Cox proportional hazards models were used to estimate hazard ratios (HRs) for mortality, stroke/TIA, and pacemaker implantation. Multivariable models adjusted for age, sex, body mass index (BMI), ischemic heart disease (IHD), hypertension, chronic kidney disease (CKD), cirrhosis, chronic obstructive pulmonary disease (COPD), atrial fibrillation (AF), diabetes mellitus (DM), peripheral artery disease (PAD), heart failure (HF), and ascending aorta diameter. HF hospitalizations were compared using Poisson regression to estimate incidence rate ratios (IRR) with 95% confidence intervals (CI). The probability of PVL was assessed using binary logistic regression.

### Age-matched analysis

To reduce age-related bias, 1:1 nearest-neighbor matching was performed by age between TAVI and SAVR patients. The matched cohort was analyzed using the same statistical methods, adjusting for sex, BMI, IHD, hypertension, CKD, cirrhosis, COPD, AF, DM, PAD, and HF. Medical records were manually reviewed to identify right bundle branch block (RBBB) at the time of the procedure, which was included in adjusted models.

### Left ventricular function and remodeling

In the matched cohort, repeated post-procedural echocardiograms obtained within 18 months were analyzed to assess left ventricular (LV) parameters. Studies were categorized into predefined intervals: baseline, 0–3, 3–6, 6–12, and 12–18 months. Repeated measures mixed-effects models accounted for within-patient correlation, and pairwise comparisons were corrected using the Holm–Sidak method.

### Sensitivity and subgroup analyses

Two sensitivity analyses were performed. First, analyses were repeated after restricting the SAVR cohort to isolated SAVR procedures, excluding patients with concomitant coronary artery bypass grafting or ascending aorta surgery. Second, within the TAVI cohort, the effect of aortic valve calcification was evaluated using pre-procedural CT-derived calcium scores, analyzed as continuous values and quartiles, for associations with mortality, pacemaker implantation, PVL, and stroke using the same multivariable model. To address the long time-frame our study spans and the advances in device technology, we performed an additional subanalysis examining the potential impact of temporal trends on our findings. Specifically, we stratified the cohort using 2018 as the cut-off point, corresponding to the introduction of the newer-generation transcatheter heart valves that remain in contemporary transfemoral use today. In addition, we compared outcomes between the two principal device platforms, self-expanding vs. balloon-expandable valves, to further account for technological heterogeneity.

All analyses were performed using R version 4.3.3 (R Foundation for Statistical Computing). A two-sided p-value <0.05 was considered statistically significant.

## Results

The final cohort included 480 patients: 405 (84%) underwent SAVR and 75 (16%) underwent TAVI. Patients treated with TAVI were significantly older and had a higher prevalence of comorbidities, including atrial fibrillation/flutter, heart failure, chronic kidney disease, and ischemic heart disease. They were also more frequently treated with ACE inhibitors or ARBs. Baseline clinical and echocardiographic characteristics are presented in Tables 1, 2. After 1:1 age matching, several baseline differences persisted, with higher rates of atrial fibrillation/flutter, heart failure, ischemic heart disease, and chronic kidney disease in the TAVI group. However, no meaningful differences were observed in baseline echocardiographic parameters (Tables 3, Supplementary Table S1).

**Table 1:** Baseline characteristics entire cohort.

Variable	Entire cohort *N* = 480	SAVR *N* = 405 (84%)	TAVI *N* = 75 (16%)
Age (SD)	62.35 (13.7)	59.9 (13)	75.9 (8)
Female (%)	171 (36)	135 (33)	36 (48)
BMI (SD)	27.75 (5.2)	27.9 (5.3)	27.0 (4.2)
Diabetes mellitus (%)	118 (24.6)	95 (24)	23 (31)
AFIB/AFL (%)	182 (37.9)	129 (32)	53 (71)
Heart failure (%)	47 (9.8)	31 (8)	16 (21)
IHD (%)	145 (30.2)	112 (28)	33 (44)
CKD (%)	53 (11.0)	29 (7)	24 (32)
HTN (%)	270 (56.2)	222 (55)	48 (64)
PAD (%)	17 (3.5)	12 (3)	5 (7)
COPD (%)	32 (6.7)	22 (5)	10 (13)
Beta blockers (%)	253 (52.7)	206 (51)	47 (63)
CCB (%)	70 (14.6)	50 (12)	20 (27)
ACEi/ARB (%)	145 (30.2)	102 (25)	43 (57)
Aorta surgery (%)	110 (23)	110 (27)	—
CABG surgery (%)	55 (12)	55 (16)	—
MAZE (%)	10 (2)	10 (2.5)	—
Implanted valve type			
Mechanical valve (%)	108 (23)	108 (27)	—
Biological valve (%)	261 (54)	261 (64)	—
Ross (%)	6 (1)	6 (2)	–
Self-expandable (%)	46 (10)	—	46 (61)
Balloon-expandable (%)	28 (6)	—	28 (37)
Missing data (%)	31 (6)	30 (7)	1 (1)

BMI, body mass index; AFIB, atrial fibrillation; AFL, atrial flutter; IHD, ischemic heart disease; CKD, chronic kidney disease; HTN, hypertension; PAD, peripheral artery disease; COPD, chronic obstructive pulmonary disease; CCB, calcium channel blockers; ACEi, angiotensin converting enzyme inhibitor; ARB, aldosterone receptor blocker; CABG, coronary artery bypass grafting.

**Table 2:** Baseline echocardiographic characteristics.

Variable	Entire cohort *N* = 480	SAVR *N* = 405 (86%)	TAVI *N* = 75 (14%)
Systolic BP mmHg (SD)	128.0 (21.3)	127.3 (21.8)	131.0 (25.0)
Diastolic BP mmHg (SD)	73.2 (12.4)	74.0 (12.1)	70.0 (12.9)
LVEF % (SD)	56.5 (11.2)	57.4 (10.3)	53.1 (14.0)
LVIVST cm (SD)	1.2 (0.3)	1.3 (0.3)	1.2 (0.2)
LVEmi g/m2 (SD)	217.7 (82.9)	206.1 (103.5)	111.3 (29.4)
AV Area Continuity Equation (SD)	0.8 (0.3)	0.8 (0.3)	0.7 (0.2)
AVmpg mmHg (SD)	46.8 (17.4)	47.1 (17.1)	45.5 (18.5)
AVppg mmHg (SD)	74.8 (25.3)	75.5 (25.6)	71.9 (23.9)
Proximal ascending aorta cm (SD)	3.9 (0.7)	3.9 (0.7)	3.7 (0.5)

BP, blood pressure; LVEF, left ventricular ejection fraction; LVIST, left ventricle interventricular septum thickness; LVEmi, left ventricle estimated mass index; AV, aortic valve SD, standard deviation; MPG, mean pressure gradient; PPG, peak pressure gradient.

**Table 3:** Baseline characteristics matched cohort.

Variable	Overall *N* = 150	SAVR *N* = 75	TAVI *N* = 75
Age (SD)	74.8 (7.0)	73.8 (6.0)	75.9 (7.8)
Female (%)	63 (42.0)	27 (36.0)	36 (48.0)
BMI (SD)	26.9 (4.6)	26.7 (5.0)	27.0 (4.2)
Diabetes mellitus (%)	46 (30.7)	23 (30.7)	23 (30.7)
AFIB/AFL (%)	88 (58.7)	35 (46.7)	53 (70.7)
Heart failure (%)	23 (15.3)	7 (9.3)	16 (21.3)
IHD (%)	62 (41.3)	29 (38.7)	33 (44.0)
CKD (%)	35 (23.3)	11 (14.7)	24 (32.0)
HTN (%)	104 (69.3)	56 (74.7)	48 (64.0)
PAD (%)	10 (6.7)	5 (6.7)	5 (6.7)
COPD (%)	13 (8.7)	3 (4.0)	10 (13.3)
Beta blockers (%)	89 (59.3)	42 (56.0)	47 (62.7)
CCB (%)	36 (24.0)	16 (21.3)	20 (26.7)
ACEi/ARB (%)	67 (44.7)	24 (32.0)	43 (57.3)
Aorta surgery (%)	15 (10)	15 (20)	—
CABG surgery (%)	13 (10.5)	3 (21)	—
MAZE (%)	1 (0.7)	1 (1.3)	—

BMI, body mass index; AFIB, atrial fibrillation; AFL, atrial flutter; IHD, ischemic heart disease; CKD, chronic kidney disease; HTN, hypertension; PAD, peripheral artery disease; COPD, chronic obstructive pulmonary disease; CCB, calcium channel blockers; ACEi, angiotensin converting enzyme inhibitor; ARB, aldosterone receptor blocker.

**Figure 2:**
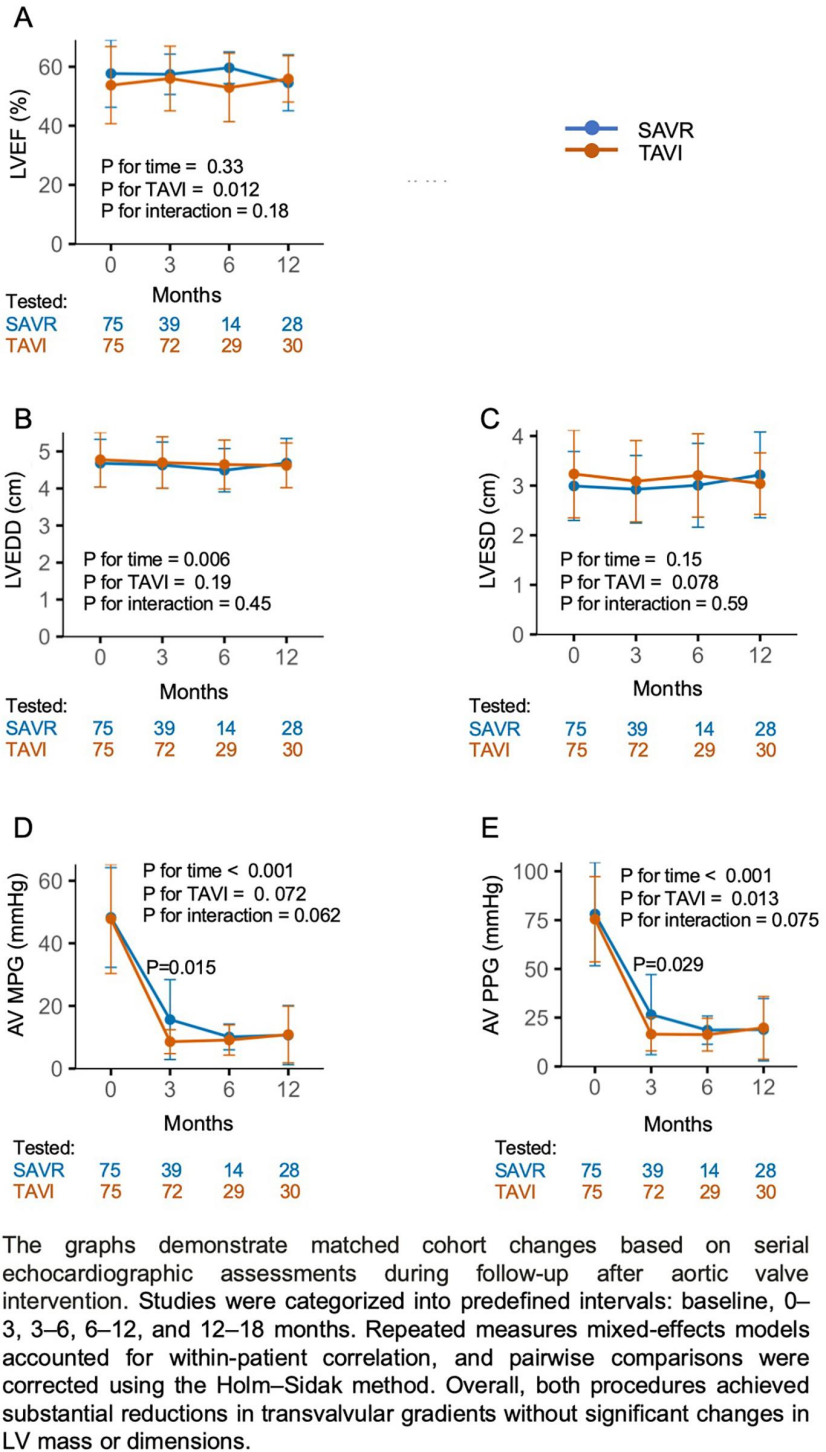
The graphs demonstrate matched cohort changes based on serial echocardiographic assessments during follow-up after aortic valve intervention. Studies were categorized into predefined intervals: baseline, 0-3, 3-6, 6-12, and 12-18 months. Repeated measures mixed-effects models accounted for within-patient correlation, and pairwise comparisons were corrected using the Holm-Sidak method. Overall, both procedures achieved substantial reductions in transvalvular gradients without significant changes in LV mass or dimensions. **(A)**: LVEF, **(B)**: LVEDD, **(C)**: LVESD, **(D)**: AV MPG, **(E)**: AV PPG. LVEF: left ventricular ejection fraction, LVESD: left ventricular end diastolic diameter, LVESD: left ventricular end systolic diameter, AV MPG: aortic valve mean pressure gradient, AV PPG: aortic valve peak pressure gradient.

Echocardiographic follow-up data were available for 111 patients (74%) in the matched cohort. Overall, no differences were observed in left ventricular ejection fraction (LVEF) before or after valve replacement. LVEF was preserved at baseline and remained stable in both groups (Figure 2A). Similarly, left ventricular end-diastolic diameter (LVEDD) and end-systolic diameter (LVESD) did not change significantly over time (Figures 2B,C). In contrast, aortic valve pressure gradients decreased markedly after valve replacement (Figure 2D). This reduction occurred earlier and was slightly greater in the TAVI group, reflected by lower peak and mean gradients at 3 months. However, by 6 months, transvalvular gradients were comparable between TAVI and SAVR (Figure 2E). Overall, both procedures achieved substantial reductions in transvalvular gradients without significant changes in LV mass or dimensions.

**Figure 3:**
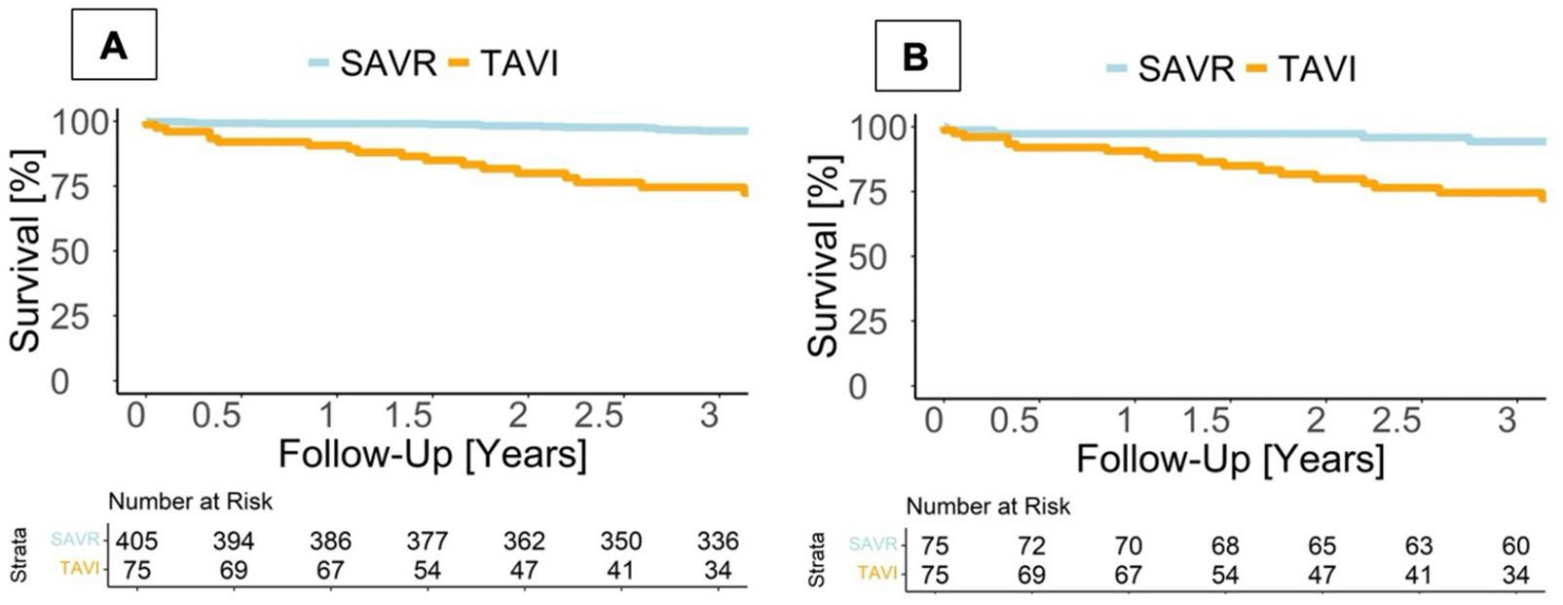
Kaplan–meier analysis. Kaplan–Meier analysis demonstrates increased mortality rates among TAVI group. Panel **(A)** Entire cohort (HR = 9.0; 95% CI, 5.4−15.0; *p* < 0.001), Panel **(B)** Matched cohort (HR = 3.6; 95% CI, 1.5−8.6; *p* = 0.004).

**Figure 4:**
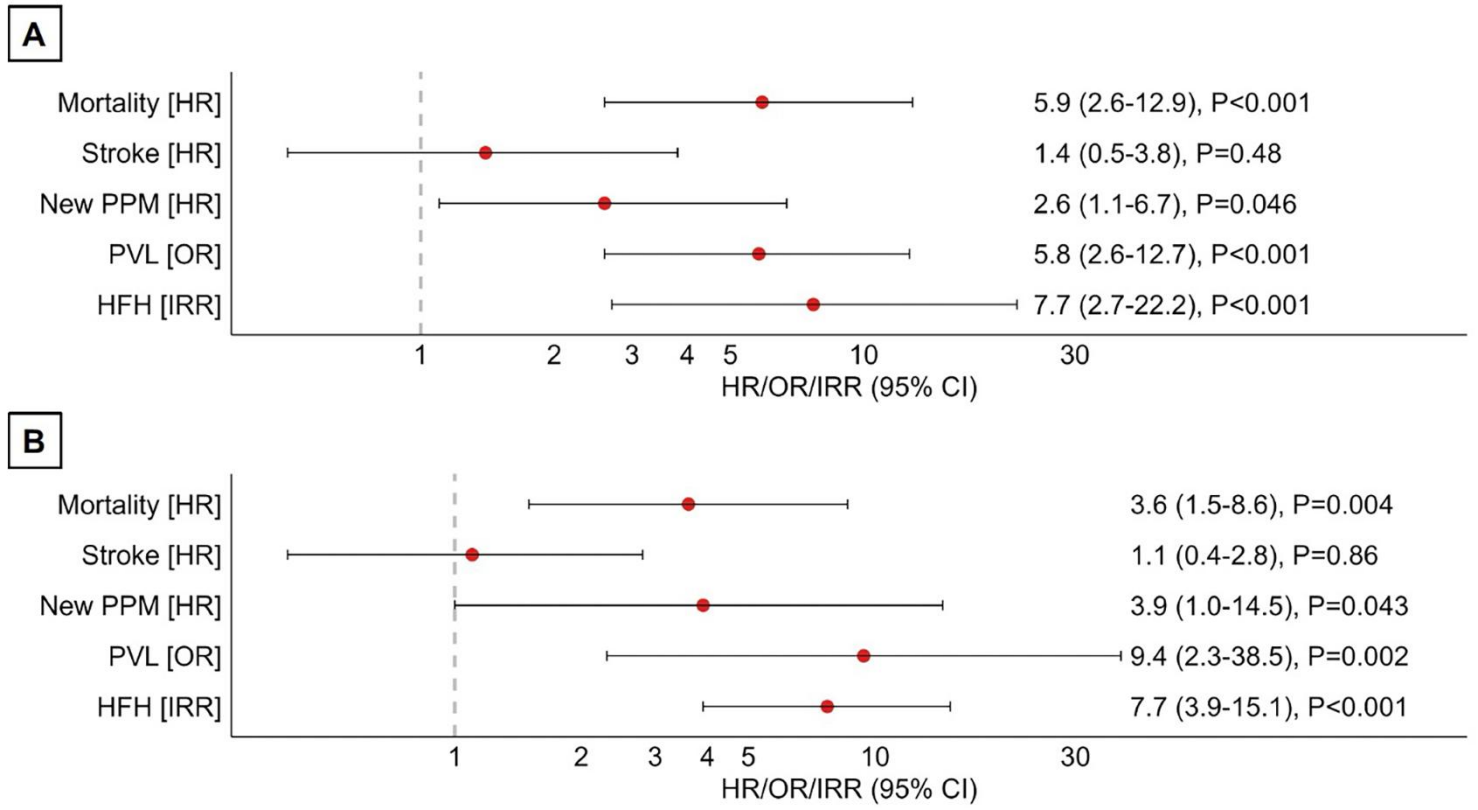
Forest plots depicting the different outcomes. The forest plots demonstrate the different outcomes according to the analyzed cohort. Panel **(A)** Adjusted analysis, Panel **(B)** Matched cohort. PPM, permanent pacemaker; PVL, paravalvular leak; HFH, heart failure hospitalization; HR, hazard ratio; OR odds ratio; IRR, incidence rate ratio.

During follow-up, 72 patients died: 28 (37%) in the TAVI group and 44 (11%) in the SAVR group. Kaplan–Meier analysis demonstrated significantly higher all-cause mortality among TAVI recipients (HR = 9.0; 95% CI, 5.4–15.0; *p* < 0.001) (Figure 3A). This association remained significant after multivariable adjustment for age and other confounders (adjusted HR = 5.9; 95% CI, 2.6–12.9; *p* < 0.001) (Figure 4A). In the 1:1 age-matched cohort, excess mortality in the TAVI group persisted (HR = 3.6; 95% CI, 1.5–8.6; *p* = 0.004) (Figure 3B).

Permanent pacemaker implantation was significantly more frequent after TAVI compared with SAVR (adjusted HR = 2.6; 95% CI, 1.1–6.7; *p* < 0.001). This association persisted in the age-matched analysis (HR = 3.9; 95% CI, 1.04–14.5; *p* = 0.043), with no interaction between baseline RBBB and procedure type (*p* = 0.84). Moderate or greater paravalvular leak (PVL) was markedly more common after TAVI (adjusted OR = 5.8; 95% CI, 2.6–12.7; *p* < 0.001) and remained significant in the matched cohort (adjusted OR = 9.4; 95% CI, 2.3–38.5; *p* = 0.002) (Figure 4B). Heart failure hospitalization rates were also significantly higher following TAVI (0.34 vs. 0.02 per patient-year; IRR = 7.7; 95% CI, 2.7–22.2; *p* < 0.001) and remained elevated in the age-matched cohort (IRR = 7.7; 95% CI, 3.9–15.1; *p* < 0.001). Rates of stroke or TIA were similar between groups (adjusted HR = 1.4; 95% CI, 0.5–3.8; *p* = 0.48), with similar findings in the matched analysis (adjusted HR = 1.1; 95% CI, 0.4–2.8; *p* = 0.86).

Sensitivity analysis restricted to isolated SAVR procedures (excluding concomitant CABG or ascending aorta surgery) yielded similar results (Supplementary Figure S1). In a separate sensitivity analysis within the TAVI cohort, the median CT-derived aortic valve calcium score was 3,057 (Q1–Q3: 2,268–3,673). Calcium score quartiles were not associated with mortality, pacemaker implantation, or PVL, but higher scores were significantly associated with increased stroke risk (Supplementary Figure S2). The results of the subgroup analyses were consistent with those of the main analysis, and the observed between-group differences persisted across procedural eras and across valve types (Supplementary Figure S3).

## Discussion

Over a 16-year period, we evaluated outcomes of patients with BAV stenosis undergoing surgical aortic valve replacement (SAVR) or transcatheter aortic valve implantation (TAVI) at our institution. SAVR was associated with significantly better long-term survival and lower rates of major complications, including permanent pacemaker implantation (PPM), paravalvular leak (PVL), and heart failure hospitalizations. These associations remained robust after adjustment for baseline clinical differences and comorbidities.

The unique anatomical features of BAV, including valve eccentricity, raphe calcification, asymmetric leaflet fusion, and frequent dilation of the ascending aorta, may create technical challenges during transcatheter procedures (4). These factors can result in incomplete prosthesis expansion, elliptical device deployment, and suboptimal annular sealing, increasing the risk of paravalvular leak (PVL) and conduction disturbances. In contrast, SAVR is less affected by these complexities because it involves complete excision of the diseased valve and implantation of a circular prosthesis under direct visualization.

Both procedures resulted in a marked reduction in aortic valve pressure gradients. TAVI showed a small early advantage in post-procedural gradients, which disappeared by 6 months. Left ventricular mass and dimensions were similar between groups and remained unchanged, consistent with prior studies showing preserved LV function after aortic valve replacement (5). While no differences were noted in trans-implantation gradients between groups, patients undergoing TAVI had a markedly higher rate of PVL. Paravalvular leak is a key determinant of long-term outcomes following aortic valve interventions. Moderate-to-severe PVL has been linked to worse survival and increased heart failure admissions (6–8). In fact, Sá et al. have suggested that even mild PVL may have adverse effects on survival and long-term outcomes in patients undergoing TAVI (9). In BAV anatomy, the elliptical annulus and heavy asymmetric calcification may contributes to incomplete sealing post-TAVI. This has been demonstrated in studies such as Yoon et al. who reported a higher incidence of PVL in BAV patients compared to those with tricuspid anatomy, and Mylotte et al., who emphasized the hemodynamic consequences of residual PVL in TAVI recipients (4, 10). Prior studies have reported conduction disturbances requiring pacemaker implantation in approximately 12% of TAVI cases, particularly in patients with bicuspid aortic valve (BAV) anatomy (11, 12). In our cohort, the rate was higher (19%), likely reflecting the older average age of our patients (76 vs. 70 years in prior studies) and the more stringent pacemaker implantation protocol at our institution.

Heart failure hospitalization (HHF) is a measure both of reduced quality of life and a marker of greater mortality (13–17). In our study, patients undergoing TAVI suffered significantly increased incidence of HHF compared with patients undergoing SAVR with an adjusted relative risk of 7.7 for the whole cohort and of 4.3 for the 1:1 age matched cohort. The markedly higher incidence rate ratio for heart-failure hospitalization observed in the TAVI group should be interpreted with caution. The TAVI cohort carried a substantially greater burden of comorbidities at baseline. Nevertheless, nationwide data from the FRANCE-TAVI registry (36,549 patients) confirmed that HF rehospitalization at 5 years occurred more frequently in those with early post-TAVI interventions including PPM placement (18). Several studies have shown that late and recurrent HF readmissions were independently associated with higher mortality (19–21). We observed no significant difference in stroke incidence between TAVI and SAVR. This finding is consistent with prior studies and large registries, including the STS/ACC TVT Registry, as well as subsequent meta-analyses demonstrating comparable stroke rates for both procedures in patients with BAV when performed at experienced centers (22).

Patients undergoing TAVI had a significantly higher risk of all-cause mortality compared with SAVR, which remained significant in the 1:1 age-matched cohort, suggesting factors beyond age and baseline risk. TAVI patients were older and had more comorbidities, which may partly explain worse survival, however the association persisted after adjustment and age matching. Procedural complications, including paravalvular leak, permanent pacemaker implantation, and possibly subclinical valve thrombosis, may also contribute to adverse long-term outcomes (23, 24). Subgroup analyses stratifying the cohort into early and late periods (according to the technological advancement) suggest that the conclusions of our study are robust to the evolution of TAVI practice over the study period and remain applicable to contemporary practice.

Our findings align with several real-world analyses. Yoon et al. reported that among patients with BAV treated with TAVI, those with severe calcification had worse outcomes, especially in terms of PVL and device success (4). Similarly, Makkar et al. found that early-generation TAVI devices were associated with increased PPM rates and worse hemodynamic performance in BAV patients compared to tricuspid anatomy (3). Several additional studies, including a meta-analysis, demonstrated similar findings with new-generation devices (25). However, other studies have shown equivalent results between TAVI and SAVR in patients with BAV stenosis. The NOTION-2 trial demonstrated that among low-risk patients under the age of 75 years with severe symptomatic AS, there were no meaningful differences in the rates of death, stroke, or rehospitalization at 1 year between TAVI and SAVR (26). These differences likely reflect the younger, lower-risk NOTION-2 population compared with our older, comorbidity-burdened real-world cohort, as well as differences in follow-up duration and device generations. Notably, NOTION-2 primarily included tricuspid aortic valves, with only one-third bicuspid valves. Nevertheless, the overall NOTION-2 results showed comparable outcomes between TAVI and SAVR, a clinically meaningful signal favouring surgery was observed within the bicuspid subgroup. Kassab et al. reported similar findings in a propensity-matched BAV cohort, supporting TAVI in selected patients (11). In sensitivity analysis, higher aortic valve calcification was associated with peri-procedural stroke but not PVL or pacemaker implantation, suggesting that calcification distribution and anatomical features may better predict outcomes and guide patient selection. An additional factor potentially contributing to the suboptimal TAVI outcomes in the bicuspid cohort is the heterogeneity of valve sizing strategies applied throughout the study period. Bicuspid anatomy, with its elliptical, often supra-annular functional plane and asymmetric calcification, predisposes to valve under-expansion and paravalvular leak when conventional annular sizing is used. Dedicated bicuspid sizing approaches have since emerged to mitigate these risks but were not uniformly adopted across centres; their progressive incorporation into contemporary practice may translate into improved outcomes.

Our study has several limitations. Its retrospective, non-randomized design introduces potential selection bias, particularly given the older, higher-risk profile of TAVI recipients. Despite multivariable and age-matched analyses, residual confounding from unmeasured factors, such as frailty, BAV subtypes (Type 0 vs. Type 1), raphe morphology, and calcification burden, may have influenced treatment allocation and outcomes. A lack of a dedicated sizing approaches for bicuspid valves are yest to be implemented universally and limits the extrapolation of our findings. Importantly, systematic data on BAV anatomical subtypes were unavailable. Finally, as a single tertiary-center study, our findings may not be generalizable to other populations.

## Conclusions

In patients with bicuspid aortic valve stenosis, SAVR was associated with better survival and lower rates of paravalvular leak, permanent pacemaker implantation, and heart-failure hospitalization compared with TAVI, while stroke and TIA rates were similar between the two treatment modalities. These findings suggest that SAVR may represent a preferable first-line strategy in selected patients with bicuspid aortic stenosis, particularly those at acceptable surgical risk. Nevertheless, given the observational nature of our study and the potential for residual confounding, TAVI remains a valuable option that should be considered on an individualized basis within a multidisciplinary Heart Team framework, taking into account surgical risk, comorbidity burden, anatomical features, and patient preference. Prospective randomized studies stratified by bicuspid aortic valve morphology are needed to more precisely define the subgroups of patients who may benefit most from a transcatheter approach.

## Data Availability

The raw data supporting the conclusions of this article will be made available by the authors, without undue reservation.
