# Supplementary material for: Surgical vs. trans-catheter aortic valve replacement in patients With bicuspid aortic valve stenosis
Source: Front Cardiovasc Med. 2026 Jun 25;13:1871325. doi: 10.3389/fcvm.2026.1871325 (PMC13346055; doi:10.3389/fcvm.2026.1871325)
Supplement: Supplementary file 1 [file Datasheet1.docx]

**Supplementary material:**

**Supplemental Table 1.** Baseline echocardiographic characteristics matched cohort

|  | Entire cohort  N=150 | SAVR  N= 75 | TAVI  N= 75 |
| --- | --- | --- | --- |
| Systolic BP mmHg (SD) | 134.1 (23.7) | 137.9 (21.4) | 130.9 (25.0) |
| Diastolic BP mmHg (SD) | 71.9 (12.4) | 74.3 (11.6) | 70.0 (12.9) |
| LVEF % (SD) | 55.1 (12.7) | 57.5 (10.4) | 53.1 (14.0) |
| LVIVST cm (SD) | 1.2 (0.2) | 1.3 (0.2) | 1.2 (0.2) |
| LVEmi g/m^2^ (SD) | 204.8 (69.4) | 202.7 (73.1) | 206.5 (66.7) |
| AV Area Continuity Equation (SD) | 0.7 (0.2) | 0.8 (0.2) | 0.7 (0.2) |
| AVmpg mmHg (SD) | 45.8 (16.9) | 46.3 (14.9) | 45.5 (18.4) |
| AVppg mmHg (SD) | 73.3 (24.2) | 75.0 (24.7) | 71.9 (23.9) |
| Proximal ascending aorta cm (SD) | 3.8 (0.6) | 3.9 (0.7) | 3.7 (0.5) |

**BP:** blood pressure, **LVEF:** left ventricular ejection fraction, **LVIST:** left ventricle interventricular septum thickness, **LVEmi:** left ventricle estimated mass index, **AV:** aortic valve **SD:** standard deviation. **MPG:** mean pressure gradient, **PPG:** peak pressure gradient.

**Supplementary figure 1.** Forest plot for sensitivity analysis of pure SAVR

**
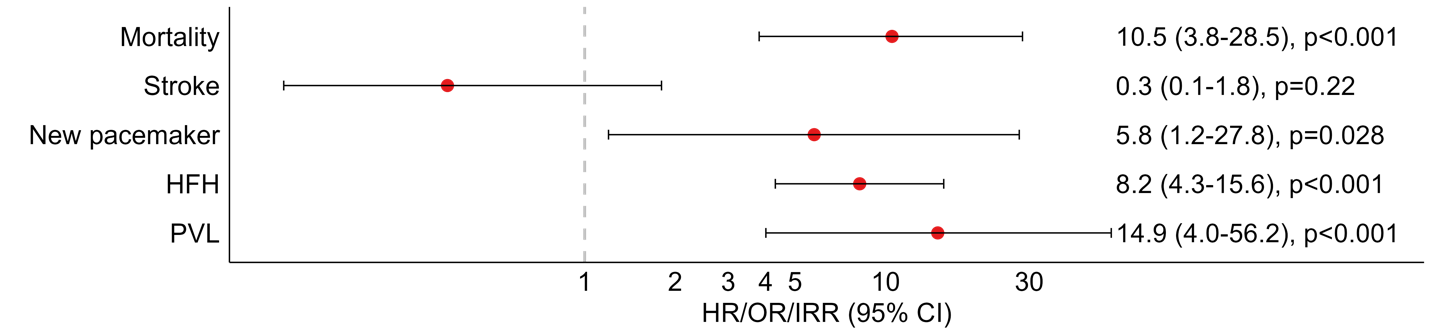
**

Sensitivity analysis to evaluate pure SAVR excluding patients that underwent combined CABG and ascending aorta surgery. Adjusted for age, sex, body mass index, ischemic heart disease, hypertension, cirrhosis, chronic kidney disease, chronic obstructive pulmonary disease, atrial fibrillation, peripheral artery disease, diabetes mellitus, heart failure, right bundle branch block, and ascending aortic diameter. **PVL:** paravalvular leak, **HFH:** heart failure hospitalizations, **HR:** hazard ratio, **OR:** odds ratio, **IRR:** incidence rate ratio.

**Supplementary figure 2.** Forest plot demonstrating the effect of calcium score on outcomes in TAVI

**
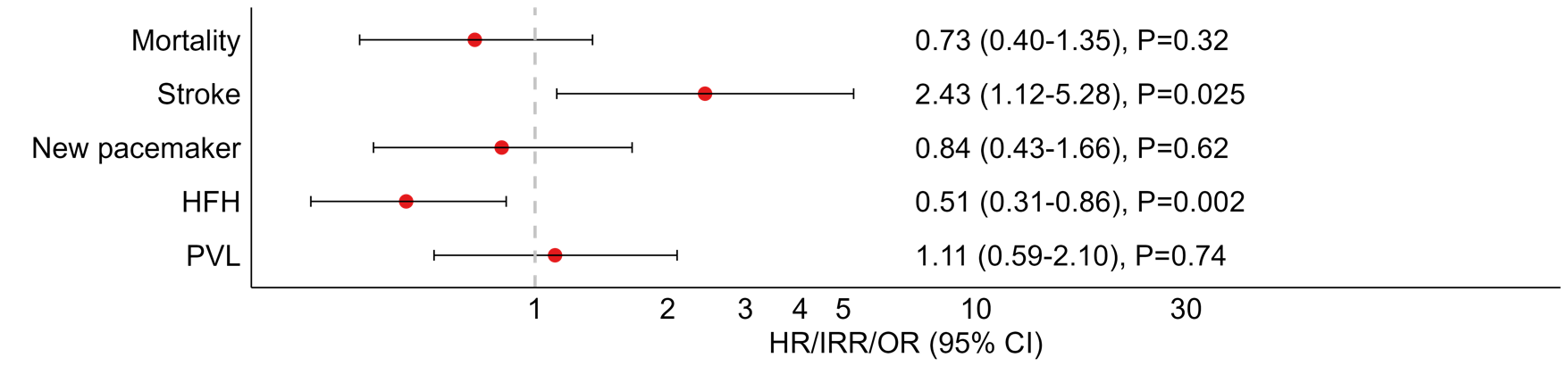
**

Sensitivity analysis in the TAVI group to evaluate the effect of the aortic valve calcification as assessed by the calcium score. Adjusted for age, sex, body mass index, ischemic heart disease, hypertension, cirrhosis, chronic kidney disease, chronic obstructive pulmonary disease, atrial fibrillation, peripheral artery disease, diabetes mellitus, heart failure, right bundle branch block, and ascending aortic diameter. **PVL:** paravalvular leak, **HFH:** heart failure hospitalizations, **HR:** hazard ratio, **OR:** odds ratio, **IRR:** incidence rate ratio.

**Supplementary figure 3.** Subgroup analyses by time period and device deployment

Stratified analysis according to TAVI era and implanted device type (balloon expanding vs. self-expandable). Evaluating the effect of the different time periods and the device type, and their impact on the outcomes. Adjusted for age, sex, body mass index, ischemic heart disease, hypertension, cirrhosis, chronic kidney disease, chronic obstructive pulmonary disease, atrial fibrillation, peripheral artery disease, diabetes mellitus, heart failure, right bundle branch block, and ascending aortic diameter. **PVL:** paravalvular leak, **PPM:** permanent pacemaker, **HFH:** heart failure hospitalizations, **HR:** hazard ratio, **OR:** odds ratio, **IRR:** incidence rate ratio.
